# Relationship between early physical activity after total knee arthroplasty and postoperative physical function: are these related?

**DOI:** 10.1186/s43019-021-00118-y

**Published:** 2021-09-28

**Authors:** Daisuke Takamura, Kentaro Iwata, Tatsuya Sueyoshi, Tadashi Yasuda, Hideki Moriyama

**Affiliations:** 1 Department of Rehabilitation, Kobe City Medical Center General Hospital, Kobe, Japan; 2 Department of Rehabilitation Science, Graduate School of Health Science, Kobe University, Kobe, Japan; 3 Department of Public Health, Graduate School of Health Science, Kobe University, Kobe, Japan; 4 Department of Orthopedic Surgery, Kobe City Medical Center General Hospital, Kobe, Japan; 5 Life and Medical Sciences Area, Health Sciences Discipline, Kobe University, Tomogaoka 7-10-2, Suma-ku, Kobe, Hyogo 654-0142 Japan

**Keywords:** Physical activity, Total knee arthroplasty, Knee osteoarthritis, Timed Up and Go test

## Abstract

**Background:**

Physical activity is associated with physical function; however, the relationship between early physical activity after total knee arthroplasty (TKA) and postoperative physical function remains unclear. The purpose of this study was to evaluate the association of early physical activity after TKA with postoperative physical function.

**Methods:**

Timed Up and Go test (TUG) of 47 patients was assessed preoperatively and at 10 days, 3 months, and 6 months postoperatively. Physical activity from the second to the ninth day after TKA was measured with accelerometer, and the correlation with pre- and postoperative physical function was evaluated . A multiple linear regression was used to predict TUG at 6 months after TKA.

**Results:**

Postoperative physical activity correlated with preoperative TUG (*ρ* = −0.485, *p* < 0.001), TUG at 10 days (*ρ* = −0.675, *p* < 0.001), 3 months (*ρ* = −0.441, *p* < 0.01), and 6 months (*ρ* = −0.368, *p* < 0.05) after surgery. Multiple linear regression indicated that only the preoperative TUG was associated with TUG at 6 months. Postoperative physical activity was not an independent factor predicting TUG at 6 months after TKA.

**Conclusion:**

Our study demonstrated that patients with better physical function have higher physical activity in the early postoperative period, whereas it does not affect physical function at 6 months after TKA. In the early postoperative period, increasing physical activity may not always be necessary to improve postoperative physical function. We also confirmed that preoperative physical function affects postoperative physical function. These findings may be beneficial in improving rehabilitation programs in the early postoperative period.

## Introduction

Physical activity is essential for decreasing the risk for mortality, chronic diseases, and lifestyle-related diseases [1, 2]. In patients with osteoarthritis, physical activity is effective to improve pain and physical function [3]. Therefore, measurement of physical activity is crucial in an aging population.

Physical activity is related to physical function in middle-aged and older adults [4]. In TKA patients, physical activity has correlated with patient-reported outcomes, such as Western Ontario and McMaster Universities Osteoarthritis Index, 36-Item Short Form Health Survey, and American Knee Society Score [5, 6]. However, little is available on the association between postoperative physical activity and physical function. Only Taniguchi et al. [7] have reported that postoperative physical activity up to 6 months was a significant predictor of improvement in Timed Up and Go test (TUG). However, it remains unclear whether early postoperative physical activity is associated with the improvement in postoperative physical function although patients stay in the hospital for a few days after TKA. Clarifying the relationship between early postoperative physical activity and postoperative physical function is critical information for improving the rehabilitation programs in the early postoperative period. The purpose of this study was to clarify the association of early physical activity after TKA with postoperative physical function. We hypothesized that physical activity in the early postoperative period is associated with postoperative physical function.

## Method

### Study design

This study was a retrospective, longitudinal observational design. The study was conducted with approval of our hospital. Baseline assessments were done 1 to 3 days preoperatively, and patients were followed up at 10 days, 3 months, and 6 months after surgery. The time point of 6 months was chosen because patients recovering from TKA typically plateau in strength and functional gains by this time point [8–10].

### Participants

Patients with knee osteoarthritis who were scheduled to undergo primary unilateral TKA in our hospital between October 2018 and August 2019 were enrolled in this study. Exclusion criteria were: (1) patients who were lost to follow-up or missing data, (2) patients who were forbidden rehabilitation because of postoperative fracture or infection, and (3) patients who had comorbidities that affect physical activity, such as musculoskeletal disorder, neurological disorder, cardiovascular disorder, psychiatric disorder, or cognitive disorder.

Patients characteristics and medical information including age, sex, height, body weight, and body mass index (BMI) were collected using clinical records.

### Surgery

All patients were operated by ten surgeons experienced in knee replacement surgery. All patients underwent cemented TKA with replacement of the patella. The medial parapatellar surgical approach was used. The inserted implant was Triathlon PS-type (Stryker), BS5 PS-type (Kyocera), ATTUNE PS-type (Depuy), or JOURNEYII PS-type (Smith & Nephew).

### Postoperative rehabilitation

All patients received the same postoperative rehabilitation protocol. In brief, patients stayed in the hospital for about 10 days. After 10 days stay in the hospital, patients were discharged to home or rehabilitation hospitals. Patients started physical therapy and full weight-bearing was allowed on the next day after surgery. Patients received continuous passive motion (CPM) for 30 min every day from the second postoperative day. The physical therapy program consisted of active or passive knee range of motion exercise, resistance training, gait exercise with or without ambulation aids, and activities of daily living training.

### Measurement of physical function

TUG was measured as the main outcome. The time in seconds was recorded for the participant to stand up from a standard chair with armrests, walk 3 m as quickly and safely as possible, turn, walk back to the chair, and sit down.

Maximal isometric knee extensor strength (KES) was measured on the bilateral legs with a hand-held dynamometer (HHD; Anima, Tokyo, Japan), as described by a previous report [11]. Participants were positioned on a platform in a sitting position with 90° hip and knee flexion, with legs perpendicular to the floor and feet not touching the ground. Sensor pads were placed on the anterior legs just proximal to the ankle joint. The length of the lever arm was measured from the estimated joint center of rotation to the center of the sensor pad. The dynamometer variable (newtons, N) and lever arm length (m) were multiplied to obtain the torque (Nm). Then, the torque value (Nm) was used to obtain the torque to body weight (Nm/kg) ratio. The strength was measured twice and the maximal value was used for the analysis.

### Measurement of physical activity

In this study, the average number of steps taken per day was used as a measure of physical activity. The physical activity was calculated using accelerometer (HJA-750C Active style Pro, Omron, Japan). The intensity of physical activity was classified into light physical activity [LPA; 1.5–3 metabolic equivalents (METs)], moderate-to-vigorous intensity physical activity (MVPA; ≥ 3 METs), and total physical activity (total PA; ≥ 1.5 METs) [12]. Participants were requested to put on the accelerometer while in the hospital, except during sleeping and bathing. Of these days, data of the first postoperative day were excluded because some patients could not put on the accelerometer on the day after surgery. Thus, we used the data from the second to the ninth postoperative days for the analysis.

### Statistical analysis

We first evaluated if the variables were normally distributed using Shapiro–Wilks test. Normally distributed variables were expressed as mean ± standard deviation, and non-normally distributed variables were expressed as median (interquartile range). To compare clinical measurements among four time points, a one-way ANOVA was used. If a significant difference was found, post hoc Bonferroni test was conducted. The correlations between clinical parameters were evaluated using Spearman’s rank correlation coefficients. Furthermore, a multiple linear regression was used to identify the predictors associated with postoperative physical function. The dependent variable was TUG at 6 months after TKA, and the independent variables were age, preoperative TUG, both sides of preoperative KES, and the average of daily step counts. The variance inflation factor (VIF) values were used to confirm multicollinearity. Data were analyzed using EZR (Saitama Medical Center, Jichi Medical University, Saitama, Japan), which is a graphical user interface for R (The R Foundation for Statistical Computing, Vienna, Austria). More precisely, it is a modified version of R commander designed to add statistical functions frequently use in biostatistics. The level of significance was set at *p* < 0.05.

## Results

**Fig. 1:**
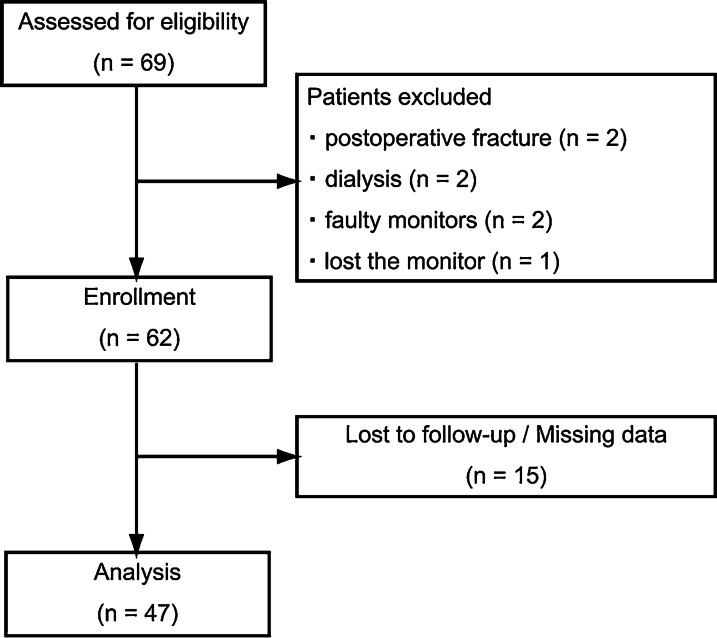
Flow chart illustrating patient recruitment from baseline to 6 months’ follow-up

In total, 69 patients underwent TKA surgery, 7 patients were excluded (2 had postoperative fracture, 2 had dialysis, 2 had faulty monitors, and 1 lost the monitor), and 15 patients were lost to follow-up; thus 47 patients were included in the final analysis (Fig. 1).

Table 1 presents the demographic characteristics of the participants. Of the 47 patients, 39 (83%) were women. The patients who completed the course had a mean age of 75.6 ± 7.7 years, height of 153.2 ± 9.2 cm, body weight of 62.9 ± 14.5 kg, and BMI of 27.0 ± 5.2 kg/m^2^.

**Table 1:** Demographic characteristics of the participants (*n* = 47)

Characteristics	Mean ± SD (%)
Age	75.6 ± 7.7
Sex	
Male/female	8 (18%)/39 (83%)
Height (cm)	153.2 ± 9.2
Body weight (kg)	62.9 ± 14.5
BMI (kg/m^2^)	27.0 ± 5.2
Side of operation	
Right/left	25 (53%)/22 (46%)
Type of implant	
Triathlon PS-type (Stryker)	20 (43%)
BS5 PS-type (Kyocera)	18 (38%)
ATTUNE PS-type (Depuy)	7 (15%)
JOURNEY II PS-type (Smith & Nephew)	2 (4%)

*SD* standard deviation, *BMI* body mass index

### Physical function and physical activity after TKA

Table 2 presents the changes in clinical measurements among four time points. TUG at 10 days after TKA was significantly slower than baseline values; however, it was significantly improved at 3 months compared with baseline values. KES on the involved side at 10 days and 3 months after TKA significantly decreased compared with baseline values, although it recovered to the preoperative values at 6 months. KES on the uninvolved side at 10 days after TKA significantly decreased compared with baseline values. There were no differences in KES on the uninvolved side at 3 months or 6 months after TKA compared with baseline values.

**Table 2:** Changes of clinical measurements among four time points

Clinical measurements	Baseline	10th postoperative day	3rd postoperative month	6th postoperative month
TUG (s)	11.03 (9.00–12.40)	15.72 (12.18–20.50)^*^	9.60 (7.90–11.50)^*,†^	8.70 (7.60–11.85)^*,†^
Involved side KES (Nm/kg)	0.81 ± 0.31	0.25 ± 0.14^*^	0.66 ± 0.23^*,†^	0.76 ± 0.20^†,‡^
Uninvolved side KES (Nm/kg)	1.01 ± 0.34	0.91 ± 0.34^§^	0.94 ± 0.29	0.94 ± 0.29

*TUG* Timed Up and Go test, *KES* knee extensor strength

^*^p < 0.01: significant difference compared with baseline value

^†^*p* < 0.001: significant difference compared with the 10th postoperative day value

^‡^*p* < 0.001: significant difference compared with the 3rd postoperative month value

^§^*p* < 0.05: significant difference compared with baseline value

**Fig. 2:**
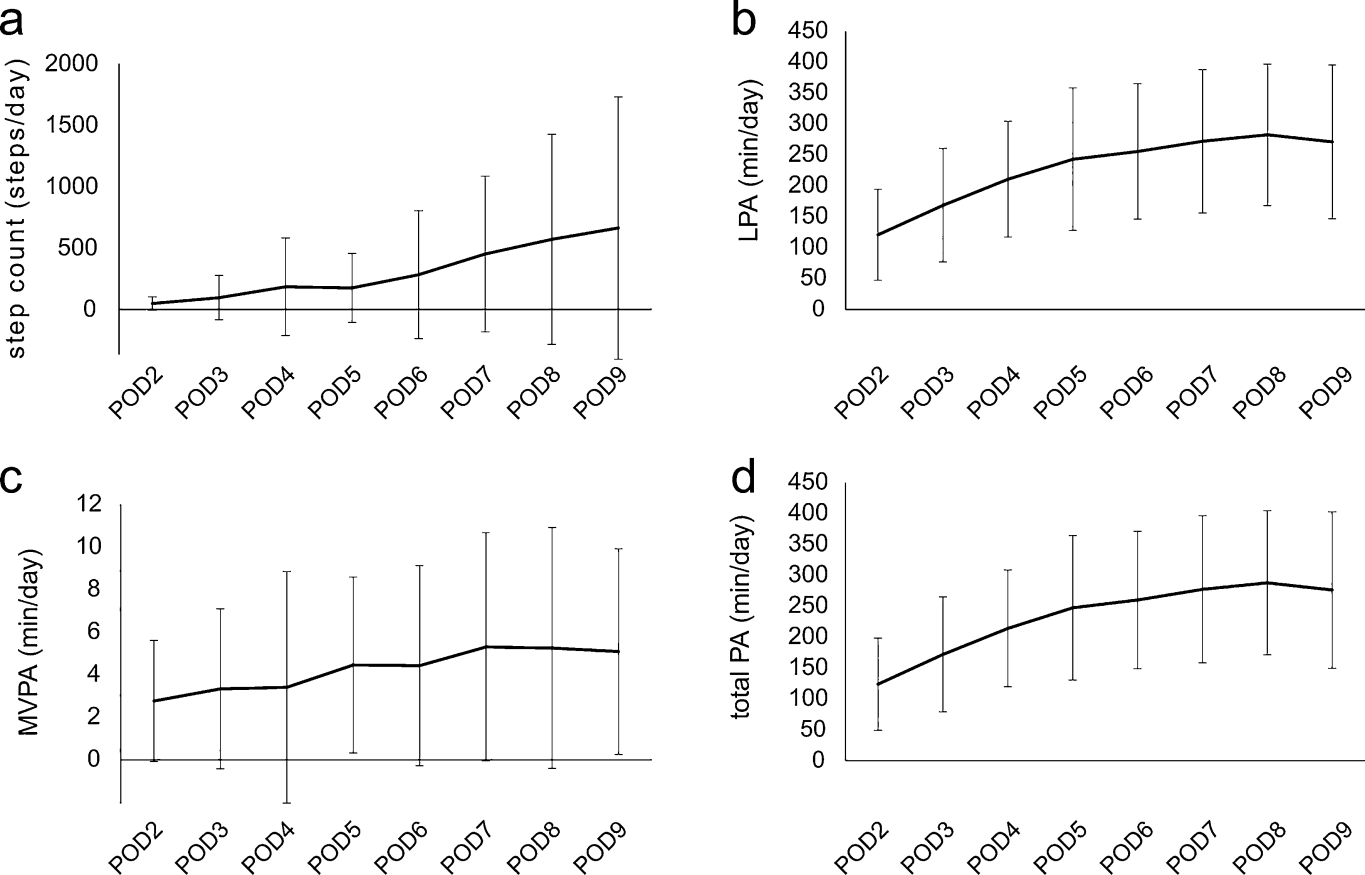
The average of **a** daily step counts and the average of daily minutes of **b** light physical activity (LPA), **c** moderate-to-vigorous intensity physical activity (MVPA), and **d** total physical activity (total PA) from the second to the ninth day after TKA with the standard deviations of the mean. Physical activity increased gradually during the hospital stay. *POD* postoperative day

Figure 2 shows the average of daily step counts and the average of daily time spent in LPA, MVPA, and total PA from the second to the ninth day after TKA. The physical activity increased gradually during the hospital stay.

### Correlation between postoperative physical activity and physical function

Table 3 presents the correlations between average of daily step counts and physical function. The average of daily step counts correlated with preoperative TUG (*ρ* = −0.485, *p* < 0.001), TUG at 10 days (*ρ* = −0.675, *p* < 0.001), 3 months (*ρ* = −0.441, *p* < 0.01), and 6 months (*ρ* = −0.368, *p* < 0.05) postoperatively.

**Table 3:** Correlation of physical activity with preoperative and postoperative TUG

Variables	Step count	
	*ρ*	*p* value
Preoperative TUG	−0.485	< 0.001
TUG at 10 days	−0.675	< 0.001
TUG at 3 months	−0.441	0.002
TUG at 6 months	−0.368	0.011

*TUG* Timed Up and Go test

### Correlation with postoperative physical function

Table 4 presents the correlations between patient demographic factors and postoperative physical function, and between preoperative and postoperative physical function. TUG at 6 months correlated with age (*ρ* = 0.486, *p* < 0.001), uninvolved side of preoperative KES (*ρ* = −0.227, *p* = 0.029), and preoperative TUG (*ρ* = 0.707, *p* < 0.001).

**Table 4:** Correlation of demographic factors with postoperative physical function

Variable	TUG at 6 months	
	*ρ*	*p* value
Age	0.486	< 0.001
BMI	0.039	0.794
Preoperative KES (involved side)	−0.227	0.125
Preoperative KES (uninvolved side)	−0.318	0.029
Preoperative TUG	0.707	< 0.001

*TUG* Timed Up and Go test, *BMI* body mass index, *KES* knee extensor strength

### Predictor of postoperative physical function

Table 5 presents the results of the multiple linear regression model for predicting TUG at 6 months. Only preoperative TUG was significantly associated with TUG at 6 months after TKA. The average of daily step counts was not associated with TUG at 6 months after TKA. These results revealed that patients who had lower values in preoperative TUG showed faster TUG times after TKA. Multicollinearity was not observed among the factors (VIF Tab = 1.11–1.47).

**Table 5:** Multiple linear regression model for predicting TUG at 6 months after TKA

Independent variable	*B*	SE	*β*	*p* value
Age	0.080	0.047	0.186	0.094
Involved side KES	0.889	1.303	0.084	0.499
Uninvolved side KES	−0.009	1.133	−0.001	0.994
Preoperative TUG	0.452	0.076	0.709	< 0.001
Step count	0.000	0.001	−0.018	0.869

*B* partial regression coefficient, *SE* standard error, *β* standardized partial regression coefficient, *KES* knee extensor strength, *TUG* Timed Up and Go test

## Discussion

We hypothesized that physical activity in the early postoperative period is an important factor for improving postoperative physical function. In contrast to our hypothesis, our study has disclosed that early postoperative physical activity is not associated with TUG at 6 months after TKA. Results of this study indicate that increasing physical activity may not always be necessary to improve postoperative physical function in the early postoperative period.

Although early postoperative physical activity correlated with postoperative TUG, multiple linear regression analysis indicated that only preoperative TUG was a significant predictor of TUG at 6 months after TKA and the average of daily step counts could not predict postoperative TUG. In the early postoperative period, inflammation such as swelling, pain, local heat, and surgical wound effusion appears in perioperative tissues. The postoperative inflammation response affects postoperative physical function such as quadriceps strength and 6-min walk test [13, 14]; therefore its management is essential. In fact, considering the postoperative inflammation, 8 days after TKA is a recommended period of time to allow maximal strength training [15]. On the other hand, progressive strength training was started within the first week after TKA [16, 17]. The appropriate timing to increase physical activity is still controversial and remains elusive. Repair of postoperative damaged tissue requires capillary growth and formation of collagen to damaged area, which takes approximately 20 days [18]. Excessive exercise that starts too early can impair the healing process of damaged tissue [18]. In the present study, we measured physical activity in the early postoperative period, when repair of damaged tissue had not been completed. Therefore, early postoperative physical activity did not affect the recovery of postoperative physical function. In a published study [19], although TKA patients underwent an intensive functional rehabilitation program during the first 2 weeks, there was no effect on the improvement of activities of daily living. Their result may support our findings that early postoperative physical activity does not affect TUG at 6 months after TKA.

Higher physical activity is typically related to better physical function [4]. In this study, although postoperative physical activity correlated with pre- and postoperative TUG, postoperative physical activity was not an independent factor predicting postoperative TUG. Considering all the results, patients with good preoperative physical function may have high physical activity in the early period after TKA and also have high physical function at 6 months after TKA. Also, patients with good preoperative physical function obtain stable ambulatory ability from the early postoperative period, thus leading to increased physical activity. From these findings, we propose that patients should maintain their physical function as high as possible before surgery. In published studies [20, 21], preoperative rehabilitation has contributed to the improvement of postoperative outcomes. These studies support our results that preoperative physical function affects postoperative physical function.

Taniguchi et al. [7] reported that postoperative physical activity up to 6 months was an independent predictor of improvement in TUG. Our results are inconsistent with their results. We measured the physical activity from the second to the ninth day after TKA, and therefore these contradictory results can be explained by a difference in time points. Collectively, we propose that physical activity beyond early postoperative period may be crucial for improving postoperative physical function. We also propose that increasing physical activity may not always be necessary to improve postoperative physical function in the early postoperative period. On the other hand, postoperative rehabilitation should be started as early as possible, because early mobilization after TKA has contributed to shortening the length of hospital stay and has decreased the incidence of postoperative deep vein thrombosis [22–24].

The present study has some limitations. First, some patients were discharged to their home directly, whereas others were admitted to rehabilitation hospitals after 10 days stay in the hospital. A systematic review [25] revealed a lack of superiority of clinic-based or inpatient rehabilitation programs compared with home-based programs in improving physical function. Although there are no data on the physical activity after discharge from the hospital, there may have been a difference between patients who were discharged to their home directly and those who were admitted to rehabilitation hospitals. Physical activity after 10 days stay in the hospital may have affected the postoperative physical function recovery, because postoperative physical activity up to 6 months is a significant predictor of improvement in TUG [7]. A second limitation is that small sample size restricted the number of variables included in the regression model. A larger sample would have allowed the inclusion of other potential predictors such as sex and comorbidity that may be associated with physical function. Studies with large numbers of participants are necessary to verify the results of the present study. Third, most of the participants were women and, therefore, whether our findings can be applied to men remains controversial. Fourth, we could not follow patients beyond 6 months postoperatively. Some studies have shown that TKA patients generally plateau in their recovery of muscle strength and physical function by 6 months after surgery [8–10]. On the other hand, some patients have had clinically significant improvement beyond 6 months postoperatively [26]. Studies with longer follow-up period are desirable. Finally, some surgeons operated on patients with various implants. Variability in surgeons and implants may have affected postoperative outcomes.

## Conclusion

Our study demonstrated that patients with better physical function have higher physical activity in the early postoperative period, whereas it does not affect physical function at 6 months after TKA. In the early postoperative period, increasing physical activity may not always be necessary to improve postoperative physical function. We also confirmed that preoperative physical function affects postoperative physical function. These findings may be beneficial in improving rehabilitation programs in the early postoperative period.

## Data Availability

The dataset of the current study is available from the corresponding author on reasonable request.

## Abbreviations

TKA: Total knee arthroplasty
TUG: Timed Up and Go test
BMI: Body mass index
KES: Knee extensor strength
LPA: Light physical activity
MVPA: Moderate-to-vigorous intensity physical activity
Total PA: Total physical activity
VIF: Variance inflation factor
